# Hydrogen Embrittlement and Failure Mechanisms in Fe–18Mn–8Al–1C–5Ni Steel with Dual B2/κ-Carbide Precipitates

**DOI:** 10.3390/ma19102137

**Published:** 2026-05-20

**Authors:** Jiahao Li, Zhilin Guo, Yuyang Qian, Xiaofei Guo, Hua Ding

**Affiliations:** 1 School of Materials Science and Engineering, Shanghai University, Shanghai 200444, China; lijiahao1223@foxmail.com (J.L.); 13929928427@163.com (Z.G.); qyy021030@163.com (Y.Q.); 2 State Key Laboratory of Advanced Special Steel, Shanghai University, Shanghai 200444, China; 3 School of Materials Science and Engineering, Northeastern University, Shenyang 110004, China

**Keywords:** Fe–Mn–Al–C–Ni steel, hydrogen embrittlement, B2 phase, κ-carbide

## Abstract

The hydrogen embrittlement (HE) behavior of an Fe–18Mn–8Al–1C–5Ni lightweight steel containing a fine and uniformly distributed B2 phase and κ-carbide was investigated by slow strain rate tensile testing with in situ hydrogen charging. Hydrogen charging reduces the elongation from 28.2% to 11.2%, while preserving an ultimate tensile strength above 1100 MPa and yielding an HE index of 60.2%. A thermal desorption analysis reveals a multi-peak desorption curve corresponding to diffusible hydrogen, hydrogen reversibly trapped at κ-carbides, and hydrogen strongly bound at the B2/γ interfaces, revealing a hierarchical hydrogen trapping behavior. Electron backscatter diffraction and electron channeling contrast imaging analyses near the fracture head region further reveal that localized hydrogen enrichment at the B2/γ boundaries induces severe stress concentration and interfacial weakening, shifting the fracture mode from ductile micro-void coalescence in air to hydrogen assisted intergranular and interphase cracking. This study clarifies the distinct roles of coherent κ-carbide and B2/γ interfaces in hydrogen trapping and crack initiation, offering a microstructure-based perspective for designing high-strength, HE resistant lightweight steels.

## 1. Introduction

Fe–Mn–Al–C austenitic lightweight steels have attracted considerable attention owing to their exceptional combination of high strength, large ductility, and reduced density [1,2,3]. These steels exhibit tensile strengths ranging from ~800 to 1500 MPa, with total elongations between ~30% and 80%, and their elemental compositions typically comprising 12–30% wt% Mn, 5–12% wt% Al, and 0.6–2.0% wt% C [3,4,5]. Mn and C stabilizes the austenitic (γ-fcc) matrix at room temperature and suppresses ferrite/martensite formation. Al provides substantial density reduction through its low atomic mass and large atomic radius, while simultaneously enabling the precipitation of ordered intermetallic phases, such as κ-carbides ((Fe,Mn)_3_AlC) and B2 (FeAl/NiAl-type) phases [3,4,5,6]. The nanoscale κ-carbides ((Fe,Mn)_3_AlCx (x < 1)) introduced during the aging treatment effectively enhance both yield strength and tensile strength without compromising ductility [7,8,9,10]. Further addition of Ni to the Fe–Mn–Al–C base system promotes the formation of ordered NiAl-type B2-structured precipitates, which provide additional precipitation strengthening through a pronounced lattice mismatch with the austenite matrix, generating strong strain fields that impede dislocation motion [11,12,13,14,15,16,17,18]. Consequently, the synergistic precipitation strengthening from the B2 phase and κ-carbide endows these alloys with exceptional specific strength [15,16]. Wang et al. [17] investigated the dynamic compressive behavior of a dual nano-precipitate-strengthened Fe–26Mn–16Al–5Ni–5C steel and concluded that significant lattice distortion arising from multi-element interactions effectively impedes dislocation motion. Our previous research [18] also revealed the excellent strain-hardening capability of an Fe–18Mn–8Al–1C–5Ni steel with a tailored fine and high-density B2 phase and nanoscale κ-carbides through controlled rolling steps. Based on calculations, the contribution of the B2 phase to the yield strength reaches 253 MPa.

Despite their attractive mechanical properties, hydrogen embrittlement (HE) remains a critical obstacle to the industrial application of high-strength austenitic lightweight steels [19,20]. As microstructural complexity increases, the mechanical and chemical interactions among the precipitate–matrix interfaces, grain boundaries, and the matrix significantly affect HE susceptibility. Several HE mechanisms have been proposed to explain the hydrogen-induced degradation, including hydrogen-enhanced decohesion (HEDE) [21], hydrogen-enhanced localized plasticity (HELP) [22], hydrogen-enhanced strain-induced vacancy formation (HESIV) [23], and hydrogen adsorption-induced dislocation emission (AIDE) [24]. Koyama et al. [25] identified grain boundary triple-junction cracking and slip-localization-assisted grain boundary cracking as dominant HE mechanisms in Fe–26Mn–11Al–1.2C (wt%) austenitic lightweight steel. Cheng et al. [26] demonstrated that κ-carbide precipitation in CoCrNi-based medium-entropy alloys impede internal hydrogen diffusion by acting as effective trapping sites, with relatively large precipitates (~50 nm) also blocking dislocation slip planes. Xiong et al. [27] reported that nanoscale κ-carbide exhibits superior HE resistance compared to coarser κ-carbide in Fe–30Mn–8Al–1.2C austenitic low-density steel. Regarding the B2 phase, Yoo et al. [28] showed that Cu-induced B2 precipitates in an Fe–0.8C–15Mn–7Al steel reduce hydrogen diffusion during deformation, while Zhang et al. [29], using density functional theory combined with thermodynamic formalism, predicted that vacancies in ordered B2 structures readily trap hydrogen atoms. Li et al. [30] further distinguished between coherent and non-coherent precipitate–matrix interfaces, stating that the former act as shallow traps while the latter function as deep traps. Unlike the Cu-induced B2 systems reported previously, the current Fe–18Mn–8Al–1C–5Ni alloy contains NiAl-type B2 precipitates together with coherent nanoscale κ-carbides in an ultrafine austenitic matrix; this dual-precipitate microstructure is expected to introduce strong B2/γ interfacial strain fields and a high density of hydrogen trapping sites with diverse trap binding energies compared to Cu-induced B2 systems or single-precipitate Fe–Mn–Al–C alloys.

Currently, reporting for the synergistic effects of the coexisting B2 phase and κ-carbide precipitates on hydrogen trapping, diffusion, and HE mechanisms remain limited in Fe–Mn–Al–C–Ni steels. In particular, the contribution of coherent κ-carbide (shallow-to-moderate traps) and non-coherent B2/γ interfaces (deep traps) to hydrogen trapping and crack nucleation has not been well addressed. Furthermore, the role of ultrafine grain size (sub-micron) in amplifying HE susceptibility through high interfacial density in these dual-precipitate systems has not been discussed.

The present study addresses these gaps by investigating the deformation and failure mechanisms of Fe–18Mn–8Al–1C–5Ni lightweight steel under hydrogen exposure. A targeted heat treatment was designed to produce finely dispersed B2 precipitates and nanoscale κ-carbides simultaneously [18]. It is hypothesized that the dual-precipitation microstructure forms a hierarchical hydrogen trapping landscape that simultaneously retard hydrogen diffusion and enhance hydrogen enrichment at precipitation–interface interfaces. To verify the role of dual precipitation on the HE resistance of the investigated material, slow strain rate tensile (SSRT) testing combined with in situ electrochemical hydrogen charging, thermal desorption spectrometry (TDS), correlative electron backscatter diffraction (EBSD) and electron channeling contrast imaging (ECCI) were employed to establish the relationship between microstructural features, particularly precipitate–material interfaces and crack initiation and propagation pathways.

## 2. Materials and Methods

### 2.1. Material and Processing

**Figure 1:**
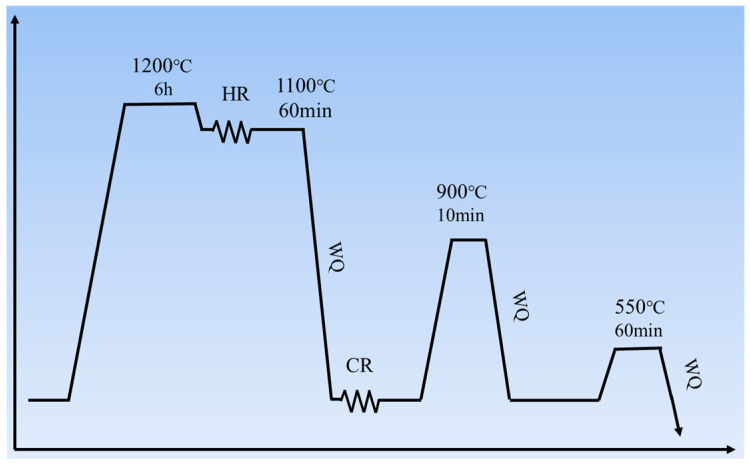
Heat treatment procedures of Fe–18Mn–8Al–1C–5Ni steel.

Steel ingots with a nominal composition of Fe–18Mn–8Al–1C–5Ni (wt%) were prepared in a vacuum induction melting furnace. They were homogenized at 1200 °C for 6 h and then immediately forged to a thickness of 70 mm, followed by hot rolling to 5 mm over five passes. Hot-rolled sheets were solution-treated at 1100 °C for 60 min and water-quenched (WQ) to obtain a fully austenitic matrix. Cold rolling (CR) was subsequently performed from 5 mm to 1 mm at 0.1 mm per pass (total reduction: 80%). Recrystallization annealing was performed at 900 °C for 10 min in inert gas and water quenched to room temperature. Subsequent aging was performed at 550 °C for 60 min in air to obtain ultrafine nanoscale κ-carbide. The detailed procedure was reported in our earlier work [18]. The complete heat treatment procedure is illustrated in Figure 1.

### 2.2. Microstructural Characterization

The material microstructure was characterized using scanning electron microscopy (SEM, Zeiss Gemini 460, Oberkochen, Germany), electron backscatter diffraction (EBSD, Oxford Instruments, Oxford, UK, step size 0.1 μm, this step size was selected to effectively distinguish the ultrafine austenitic grains), electron channeling contrast imaging (ECCI), transmission electron microscopy (TEM; Thermo Fisher Talos F200X, Waltham, MA, USA), and energy dispersive spectrometer (EDS) elemental maps. EBSD specimens were prepared by mechanical polishing with subsequent vibratory polishing with a 0.05 μm colloidal silica suspension for 6 h. Grain size was quantified using the equivalent circle diameter method based on EBSD data. TEM specimens were mechanically thinned to approximately 50 μm and then electro-polished by twin-jet polishing in a 10 vol.% perchloric acid–ethanol solution at 20 V and −20 °C.

### 2.3. Evaluation of Hydrogen Embrittlement Sensitivity

**Figure 2:**
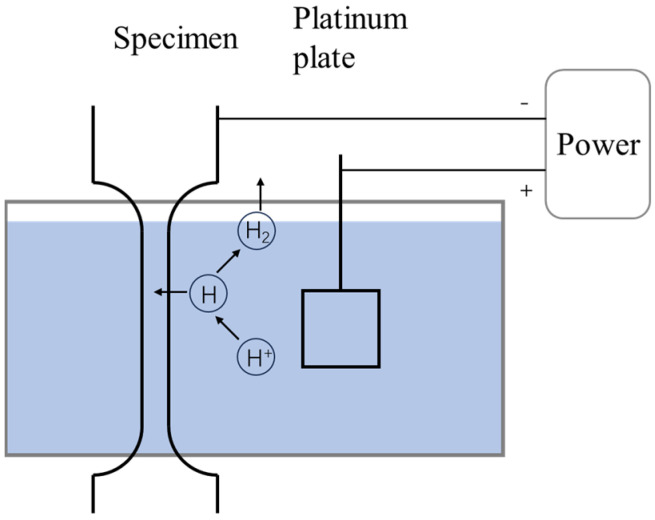
Schematic diagram of an in situ hydrogen charging device.

SSRT testing with tensile specimens (gauge dimensions of 25 × 5 × 1 mm^3^, aligned along the rolling direction) was performed at a strain rate of 10^−5^ s^−1^ at room temperature using universal tensile testing equipment (LETRY WDML-50, Xi’an, China). HE susceptibility was evaluated by in situ electrochemical hydrogen charging during SSRT testing at the same strain rate under cathodic hydrogen charging in 3 wt% NaCl + 0.3 wt% CH_4_N_2_S at a current density of 5 mA/cm^2^. At least two repeated tests were performed for each condition. A schematic diagram of the in situ hydrogen charging device is shown in the Figure 2.

The hydrogen embrittlement index (HEI) is calculated using the following formula:

(1) $$HEI=\frac{EL_{0}-EL_{H}}{EL_{0}}$$

where *EL*_0_ is the total elongation of the specimen in air conditions, and *EL_H_* is the total elongation under in situ hydrogen charging conditions.

The specimens were cleaned in ethanol and stored in liquid nitrogen directly after the SSRT tests had finished. The hydrogen content and thermal desorption behaviors were investigated by hydrogen mass spectroscopy (Bruker Galileo 8, Ettlingen, Germany) at a heating rate of 20 °C/min from room temperature to 800 °C. Deconvolution on the TDS curve using Gaussian-only fitting was performed to separate individual peaks, to identify discrete desorption events, and to estimate the hydrogen content associated with each trap state.

## 3. Results

### 3.1. Microstructure

**Figure 3:**
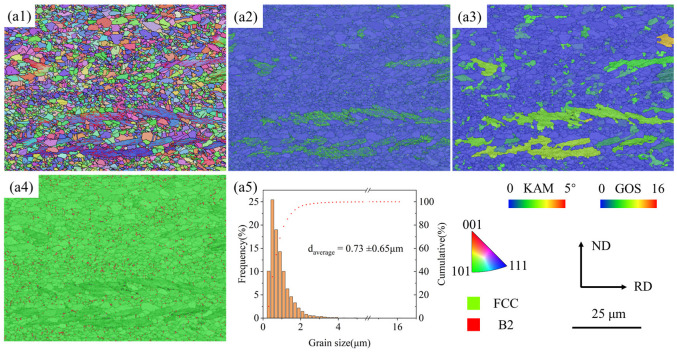
EBSD microstructure characterization: (**a1**) IPF + GB map, (**a2**) KAM map, (**a3**) GOS map, (**a4**) Phase distribution map, (**a5**) Grain size distribution map.

The initial microstructure of the investigated steel is presented in Figure 3. The inverse pole figure and grain boundary (IPF + GB) distribution map in Figure 3a1 reveals that the matrix contains ultrafine equiaxed grains with a small amount of banded grains elongated along the rolling direction, indicating incomplete recrystallization at these locations. The kernel average misorientation (KAM) map (Figure 3a2) shows low KAM values (0.15°) in the recrystallized microstructure, and elevated values (1.03°) at the banded structure. The grain orientation spread (GOS) map (Figure 3a3) displays that the regions with GOS > 2° account for approximately 28.4% of the mapped area, indicating substantial deformed regions with existing dislocation networks. The phase distribution map (Figure 3a4) reveals the NiAl-type B2 phase distributed mostly along the austenite grain boundaries and triple junctions. The austenite has an average grain size of 0.73 ± 0.65 μm, as statistically analyzed in Figure 3a5.

**Figure 4:**
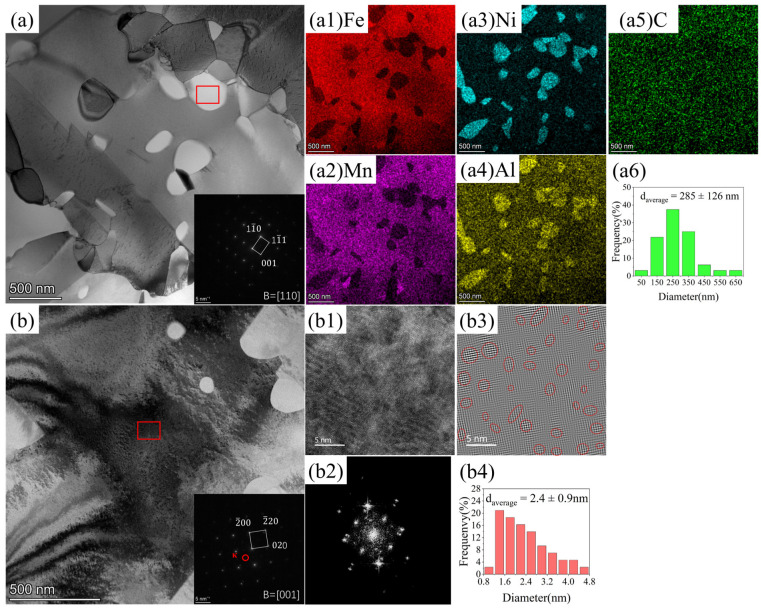
TEM microstructure characterization: (**a**) BF micrograph with SAED patterns from the marked region; (**a1**–**a5**) EDS maps corresponding to (**a**); (**a6**) The diameter distribution of the B2 phase; (**b**) BF micrograph and SAED patterns from the marked region; (**b1**) HRTEM image of the austenitic matrix; (**b2**) FFT image corresponding to (**b1**); (**b3**) IFFT image corresponding to (**b2**), with red circles indicating κ-carbide; (**b4**) The diameter distribution of κ-carbides.

TEM characterization was performed to further elucidate the precipitate characteristics in Figure 4. The bright-field (BF) TEM image (Figure 4a) shows that the B2 precipitates are dispersed both at the grain boundaries and in the interior, showing an irregular morphology. The selected area electron diffraction (SAED) pattern acquired from the boxed region along the B = [110] zone axis displays diffraction spots consistent with a body-centered cubic (BCC) superlattice structure, confirming the B2 crystal structure. Furthermore, EDS elemental maps (Figure 4a1–a5) confirm that the B2 phase is enriched in Ni and Al (Figure 4a3,a4) with corresponding depletion in Fe and Mn (Figure 4a1,a2), while C distribution (Figure 4a5) is uniform with no notable enrichment in the B2 regions.

The BF TEM image (Figure 4b) further reveals a high number density of nanoscale precipitates within the austenitic matrix, dispersed uniformly and forming local clusters. The SAED pattern from the marked region along the B = [001] zone axis shows superlattice reflections characteristic of the ordered κ-carbide phase in addition to the fundamental austenite diffraction spots, indicating a cube-on-cube orientation relationship: [100]κ//[100]γ and (100)κ//(100)γ. The high-resolution TEM (HRTEM) image (Figure 4b1) shows continuous lattice fringes of the austenite matrix. The corresponding Fast Fourier Transform (FFT) pattern (Figure 4b2) exhibits frequency features associated with ordered precipitates, and the inverse FFT (IFFT) image (Figure 4b3), and the pattern obtained by filtering for the superlattice spots reveals nanoscale ordered domains (highlighted by red circles), confirming that κ-carbide exists as coherent nanoscale precipitates.

The HRTEM image (Figure 4b1) displays continuous lattice fringes of the austenite matrix. The corresponding Fast Fourier Transform (FFT) pattern (Figure 4b2) exhibits frequency features associated with ordered precipitates. In the Inverse Fast Fourier Transform (IFFT) image (Figure 4b3), generated by filtering the FFT pattern, nanoscale ordered domains are clearly visible, as highlighted by red circles. Nanoscale κ-carbides distribute dispersedly in the matrix with coherency to the parental matrix. Based on a statistical analysis, the B2 phase has the average diameter of 285 ± 126 nm and a volume fraction of 13.9%, while the κ-carbides have an average diameter of 2.4 ± 0.9 nm and a volume fraction of 14.3%.

### 3.2. Hydrogen Desorption Behaviors

**Figure 5:**
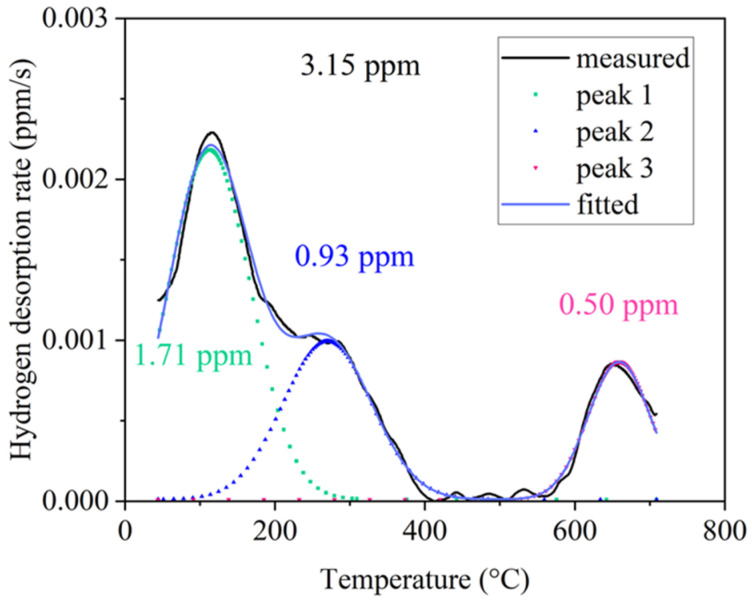
Hydrogen desorption curves with Gaussian curve deconvolution into individual peaks.

The TDS analysis results following 24 h of electrochemical pre-charging are presented in Figure 5. Based on the deconvolution of the TDS curve, three apparent desorption peaks can be distinguished, corresponding to a total measured hydrogen content of approximately 3.15 ppm. Peak 1 (center temperature ~112 °C; ~1.71 ppm) could be associated with diffusible hydrogen released from interstitial solid-solution sites within the austenite matrix and from shallow traps, such as vacancies and dislocations [31]. Peak 2 (center temperature ~269 °C; ~0.93 ppm) is likely related to hydrogen trapped at moderate-strength reversible trapping sites. Considering the high volume fraction and interface density of coherent κ-carbides, these precipitates may contribute substantially to the intermediate-temperature desorption process, which is broadly consistent with the ab initio predictions of moderate hydrogen–carbide binding energies in similar alloy systems [26,32]. Peak 3 (center temperature ~659 °C; ~0.50 ppm) is tentatively assigned to hydrogen in deep, high-energy traps probably associated with the B2 phase, particularly at non-coherent B2/γ interfaces where compositional discontinuities and lattice strain fields provide strong binding sites [29,30]. The markedly higher desorption temperature of Peak 3 suggests the presence of strongly trapped hydrogen with limited mobility at room temperature; therefore, this fraction may be less likely to participate directly in hydrogen transport coupled with dislocation motion during room-temperature SSRT testing. Overall, the multi-peak desorption profile suggests a hierarchical hydrogen-trapping scheme introduced by the dual-precipitate microstructure: the austenite matrix may provide shallow interstitial and defect-related trapping sites, κ-carbide may introduce moderate-strength trapping sites, and the B2/γ interface network may contribute to deep hydrogen trapping sites.

### 3.3. Hydrogen Embrittlement Sensitivity

**Figure 6:**
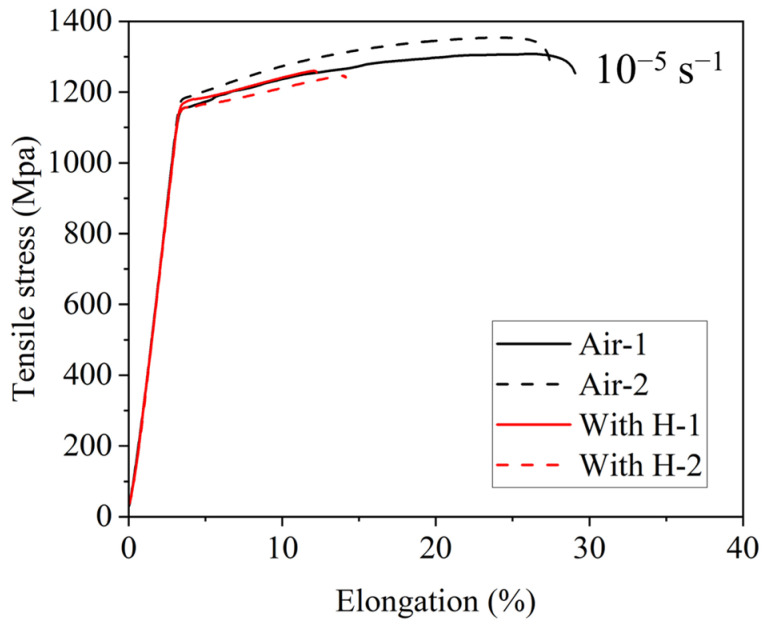
SSRT tensile stress–elongation curves of Fe–18Mn–8Al–5Ni–1C steel in air and in situ hydrogen charging conditions.

The engineering stress–strain curves under uncharged (air) and in situ hydrogen-charged conditions are presented in Figure 6. The uncharged specimen exhibits an average yield strength (YS) of 1162 ± 14 MPa, an average ultimate tensile strength (UTS) of 1331 ± 23 MPa, and a total elongation (EL) of 28.2 ± 0.8%. Under in situ hydrogen charging, the YS remains at 1168 ± 7 MPa and the UTS decreases slightly to an average of 1253 ± 8 MPa. By contrast, the EL decreases markedly to 11.2 ± 1.0%, yielding an HEI of 60.2%. These results confirm that hydrogen charging induces pronounced ductility deterioration with minimal effects on strength, indicating high HE susceptibility of this alloy.

### 3.4. Fracture Surface Morphology and Crack Propagation Pathway

**Figure 7:**
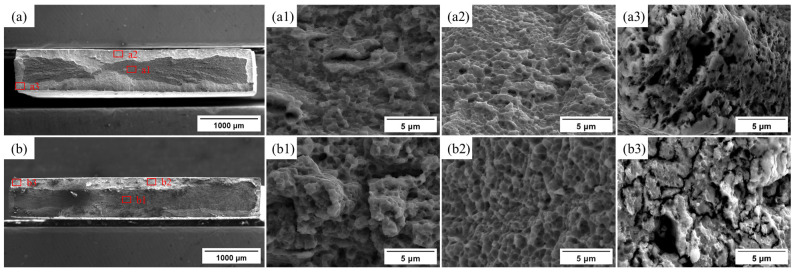
SEM images of tensile specimens tested (**a**,**a1**–**a3**) in air and (**b**,**b1**–**b3**) under in situ electrochemical hydrogen charging.

The fracture surface characterization of the specimens tested in air and under in situ hydrogen charging is presented in Figure 7. The uncharged specimen (Figure 7a) exhibits prominent macroscopic necking and shear lips, indicating a ductile fracture dominated by extensive plastic deformation. Higher-magnification observation (Figure 7a1) reveals that, among dense fine dimples with micro-void coalescence, some banded deformation zones exhibit transverse elongated dimples, probably correlated to the non-recrystallized regions. The ultrafine B2 phase and κ-carbide would serve as micro-void nucleation sites during deformation. By comparison, the hydrogen-charged specimen (Figure 7b) displays a flatter fracture surface with limited necking behavior. Higher magnification (Figure 7b1) images revealed some localized quasi-cleavage regions, indicating interfacial degradation probably due to hydrogen enrichment at austenite/B2 boundaries. Further analysis of the local high-magnification morphology at the fracture corner reveals that the air-exposed tensile specimen (Figure 7a3) still exhibits relatively prominent dimples and tear ridges, indicating that, even at the localized stress-concentrated corner of the fracture, the material can still achieve stress relief through sufficient plastic deformation, with its fracture mechanism primarily dominated by ductile fracture through micro-void nucleation, growth, and coalescence. By contrast, the hydrogen-charged specimen (Figure 7b3) shows a tendency toward shallow and small dimples, accompanied by the presence of numerous local secondary cracks, indicating that hydrogen significantly weakens the plastic coordination capability at these sites, promoting crack initiation and rapid propagation in the stress-concentrated region. This result demonstrates that hydrogen has a particularly pronounced embrittling effect on these sites, causing the local fracture mechanism to transition gradually from ductile micro-void coalescence to a quasi-cleavage-dominated brittle fracture. Consequently, fracture in air is dominated by micro-void coalescence, whereas in situ charged specimens reveal increasing quasi-cleavage regions, accompanied by increased secondary cracking and reduced necking.

**Figure 8:**
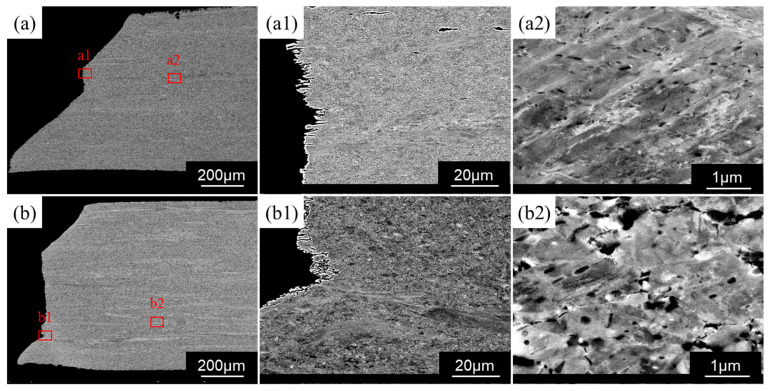
ECCI of the longitudinal cross-sections at the fracture head regions of SSRT specimens tested: (**a**,**a1**,**a2**) in air and (**b**,**b1**,**b2**) under in situ hydrogen charging conditions.

The ECCI of fracture cross-sections under both conditions are presented in Figure 8. The specimen tested in air exhibits larger thickness reduction in the fracture head region (Figure 8a), with secondary cracks aligned predominantly parallel to the tensile direction in the central zone (Figure 8a1). In the homogenized deformed region (Figure 8a2), microbands with an average width of 0.12 μm and surface density of 0.027 μm^−2^ are observed (quantified over a 20 × 20 μm^2^, representative area using ImageJ^®^ (Version: 2.14.0) threshold segmentation), reflecting coordinated multi-slip dislocation activity during tensile deformation.

The specimens tested under in situ charging conditions (Figure 8b) exhibited a significantly reduced necking, indicating diminished macroscopic plasticity. As shown in Figure 8b1,b2, the microbands are distinctly less than the unchanged condition.

Under ambient air conditions, crack propagation is primarily strain-controlled. Plastic deformation at the crack tip promotes crack blunting and redistributes micro-band coordination strains. Oriented secondary cracks reflect stress-guided growth along tensile axes. Under hydrogen charging, propagation transitions to brittle-dominated with networked cracks and increased branching.

**Figure 9:**
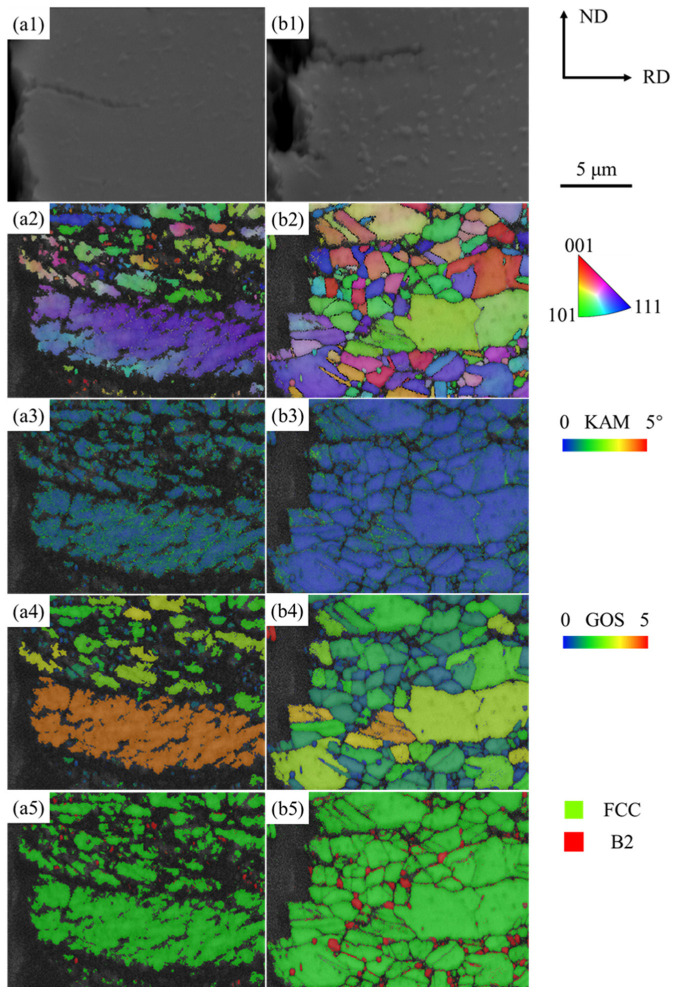
EBSD characterization of cracks in the central brittle region of the fracture longitudinal section of tensile specimens: (**a1**–**a5**) tested in air; (**b1**–**b5**) under in situ hydrogen charging. (**a1**,**b1**) SEM images, (**a2**,**b2**) IPF + GB maps, (**a3**,**b3**) KAM maps, (**a4**,**b4**) GOS maps, (**a5**,**b5**) Phase distribution maps.

The EBSD patterns at crack locations within the central brittle zone of fracture longitudinal cross-sections are presented in Figure 9. Figure 9a1,b1 show the location of micro-cracks in the uncharged and hydrogen-charged specimens, respectively. Figure 9a2 shows secondary cracks propagating along the interface between the interfaces of recrystallized grains and unrecrystallized bands in the specimens tensile-deformed in air. Figure 9a3 reveals a broader region of higher KAM (averagely 0.8°) in the crack vicinity in the unrecrystallized bands, indicating that the pronounced deformation strain suppresses further material flow and initiates interface debonding. The location of the unrecrystallized bands is confirmed by the high GOS value (Figure 9a4), in which the B2 phases were not observed (Figure 9a5).

In the hydrogen-charged condition, the crack propagates in the recrystallized region, preferentially through the grain boundaries between the B2 and austenite matrix (Figure 9b2). The KAM values (averagely 0.45°) are generally low (Figure 9b3), with higher KAM values beneath the grain boundaries. It indicates that strain concentrates at the grain boundaries and promotes damage initiation, although the grains have low GOS values (Figure 9b4). The B2 phases are assumed to be closely related to the crack propagation routine, as indicated by the reduced image quality around the B2 phases (Figure 9b5).

## 4. Discussion

### 4.1. The Influence of Hydrogen on Deformation Mechanisms and Strain Distribution

The uncharged material exhibits a pronounced strain hardening behavior with excellent plasticity through the intermetallic B2 phase, κ-carbide precipitation, as well as microbanding induced plasticity, which has also been reported in our previous work [18]. Microbands formed through dislocation slip in the austenitic matrix promote multi-slip coordination and dislocation storage, effectively facilitating strain redistribution and delaying local instability [33,34,35]. This coordinated strengthening mechanism in the recrystallized microstructure mitigates the mechanical incompatibility with the unrecrystallized banded structure until cracks are initiated at these heterogeneous boundaries at high deformation strains (Figure 9a1–a5).

By comparison, the hydrogen-charged specimens revealed a pronounced reduction in ductility, as shown in Figure 6. Their fracture surfaces appear relatively flat with negligible necking (Figure 7 and Figure 8). As shown in the fracture head region in Figure 8b2, the micro-banding is scarcely developed in the region adjacent to the fracture surfaces. This morphological evolution suggests that the microband-mediated plastic accommodation, which dominates the deformation of the hydrogen-free counterpart, is largely suppressed under hydrogen charging. Such suppression is consistent with the reported work on hydrogen promoting dislocation planarity and inhibiting cross-slip in Fe–Mn–C systems [36], a phenomenon commonly attributed to the hydrogen-enhanced localized plasticity (HELP) mechanism. In the presence of the hard intermetallic B2 phase, such planar dislocation arrays are expected to pile up against B2/γ interfaces, generating pronounced local triaxial stresses that further drive diffusible hydrogen toward these interfaces. The resulting local hydrogen enrichment progressively reduces the cohesive strength of the B2/γ interfaces, thereby activating the HEDE mechanism [32,37]. With continuous hydrogen uptake, micro-voids preferentially nucleate at these embrittled interfaces, coalesce into a networked crack pattern, and propagate rapidly along the grain and phase boundaries [23], ultimately producing a flat fracture surface with substantial brittle features.

### 4.2. Hydrogen Trapping Behaviors and the Resultant Crack Initiation and Propagation

Combining microstructural observations with TDS multi-peak desorption results, the hydrogen trapping behavior of the dual-precipitation system can be proposed. The high volume fraction (14.3%) of nanoscale κ-carbides (~2.4 nm, full coherent with austenite matrix) possess both high interface density and localized coherency strain fields [38]. These κ-carbides may act as distributed moderate-strength trapping sites (Peak 2, ~269 °C). Owing to their fine size, coherent nature, and relatively uniform distribution within the austenitic matrix, these κ-carbides are more likely to affect hydrogen redistribution and to reduce long-range hydrogen transport, rather than directly serving as dominant crack-initiation sites. Therefore, their influence on HE is likely less adverse than that of localized interfacial traps, although their contribution to the overall hydrogen-trapping capacity remains important.

**Figure 10:**
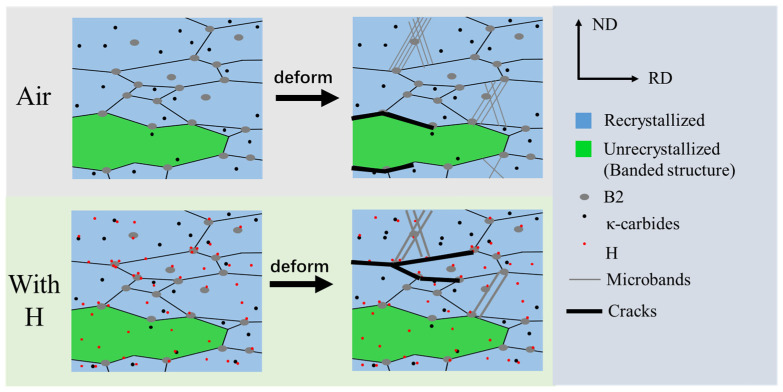
Schematic diagram of crack propagation path.

The B2 phase, partially located at the grain boundaries with pronounced compositional segregation (Ni- and Al-enriched), generates both strain field and chemical discontinuities at the B2/austenite interface. The high-temperature desorption peak is preliminarily attributed to deep traps related to the B2/γ interface (Peak 3, ~659 °C) and simultaneously as preferential crack initiation sources under applied loading [29,39]. In the in situ hydrogen charging condition, the cohesion force at the B2/γ interfaces is reduced by the locally elevated hydrogen concentration (HEDE), facilitating premature interfacial separation and crack nucleation. It is assumed that the interplay of pre-existing hydrogen occupation at these high-energy sites and the stress-assisted redistribution of nearby diffusive hydrogen may promote hydrogen-enhanced decohesion at the B2/γ interface [40,41,42]. The schematic diagrams of crack propagation paths under two tested conditions are shown in the Figure 10.

In summary, the hierarchical hydrogen-trapping behavior plays a key role in connecting local deformation with final fracture. The diffusive hydrogen in shallow traps in the austenitic matrix and the dislocations and interfaces of coherent κ-carbides may diffuse to high stress concentration regions formed during deformation. The hard B2 phases located at the grain boundaries and triple junctions would act as preferential sites of local stress concentration, which in turn drives sustained hydrogen accumulation until the critical condition for crack initiation is reached. As a result, hydrogen first promotes localized deformation through HELP, and then weakens the hydrogen-enriched B2/γ interfaces through HEDE, finally shifting the crack path from strain-controlled cracking at the unrecrystallized/recrystallized interfaces in air to hydrogen-assisted interfacial cracking along the B2/γ boundaries.

## 5. Conclusions

This study investigates the microstructural characteristics and hydrogen embrittlement behavior of Fe–18Mn–8Al–5Ni–1C austenitic lightweight steel containing a dual B2/κ-carbide precipitate microstructure. The main conclusions are as follows:

1. The microstructure consists of an ultrafine austenitic matrix (average grain size 0.73 μm) containing NiAl-type B2 precipitates (average diameter 285 nm, volume fraction 13.9%) and coherent nanoscale κ-carbides (average diameter 2.4 nm, volume fraction 14.3%). The dual precipitates in different scales induces exceptional strain hardening.
2. The TDS analysis reveals that the κ-carbide and B2/γ interfaces act as moderate and high energy hydrogen traps, respectively, generating a multi-peak desorption profile. The diffusible and reversibly trapped hydrogen (Peaks 1 and 2, ~2.64 ppm) can be redistributed to stress the concentrated B2/γ interfaces, where local hydrogen enrichment progressively weakens interfacial cohesion and ultimately triggers interfacial cracking along the B2/γ boundaries. The results suggest the important role of the B2/γ interfacial traps in local damage evolution under coupled hydrogen-stress conditions.
3. Hydrogen charging induces pronounced ductility deterioration (EL reduced from 28.2% to 11.2%; HEI = 60.2%). The in situ hydrogen charging leads to deformation localization at the B2/austenite interfaces. The crack propagation pathways shift from the interfaces between the banded deformation structure and recrystallized grains in the uncharged condition to the B2/austenite interface decohesion upon hydrogen exposure.

The present findings demonstrate that tailoring the distribution and interfacial characteristics of the B2 precipitates and nanoscale κ-carbides enables the regulation of hierarchical hydrogen trapping and localized plastic deformation, providing a microstructure design strategy for developing high-strength, HE resistant lightweight steels.

## Data Availability

The original contributions presented in this study are included in the article. Further inquiries can be directed to the corresponding authors.

## Abbreviations

The following abbreviations are used in this manuscript:

HE	Hydrogen embrittlement
SSRT	Slow strain rate tensile
TDS	Thermal desorption spectroscopy
EBSD	Electron backscatter diffraction
ECCI	Electron channeling contrast imaging
HEDE	Hydrogen-enhanced decohesion
HELP	Hydrogen-enhanced localized plasticity
HESIV	Hydrogen-enhanced strain-induced vacancy formation
AIDE	Hydrogen adsorption-induced dislocation emission
SEM	Scanning electron microscopy
TEM	Transmission electron microscopy
EDS	Energy dispersive spectrometer
HEI	Hydrogen embrittlement index
IPF	Inverse pole figure
GB	Grain boundary
KAM	Kernel average misorientation
GOS	Grain orientation spread
BF	Bright-field
SAED	Selected area electron diffraction
BCC	Body-centered cubic
HRTEM	high-resolution TEM
FFT	Fast Fourier transform
IFFT	Inverse FFT
YS	Yield strength
UTS	Ultimate tensile strength
EL	Total elongation
